# Geraniin Protects High-Fat Diet-Induced Oxidative Stress in Sprague Dawley Rats

**DOI:** 10.3389/fnut.2018.00017

**Published:** 2018-03-16

**Authors:** Alexis Panny Y. S. Chung, Sunil Gurtu, Srikumar Chakravarthi, Mohanambal Moorthy, Uma D. Palanisamy

**Affiliations:** ^1^School of Medicine and Health Sciences, Monash University Malaysia, Sunway City, Malaysia; ^2^Department of Pathology, Perdana University, Serdang, Malaysia

**Keywords:** geraniin, high-fat diet, oxidative stress, type-2 diabetes, rats

## Abstract

Geraniin, a hydrolysable polyphenol derived from *Nephelium lappaceum* L. fruit rind, has been shown to possess significant antioxidant activity *in vitro* and recently been recognized for its therapeutic potential in metabolic syndrome. This study investigated its antioxidative strength and protective effects on organs in high-fat diet (HFD)-induced rodents. Rats were fed HFD for 6 weeks to induce obesity, followed by 10 and 50 mg/kg of geraniin supplementation for 4 weeks to assess its protective potential. The control groups were maintained on standard rat chows and HFD for the same period. At the 10th week, oxidative status was assessed and the pancreas, liver, heart and aorta, kidney, and brain of the Sprague Dawley rats were harvested and subjected to pathological studies. HFD rats demonstrated changes in redox balance; increased protein carbonyl content, decreased levels of superoxide dismutase, glutathione peroxidase, and glutathione reductase with a reduction in the non-enzymatic antioxidant mechanisms and total antioxidant capacity, indicating a higher oxidative stress (OS) index. In addition, HFD rats demonstrated significant diet-induced changes particularly in the pancreas. Four-week oral geraniin supplementation, restored the OS observed in the HFD rats. It was able to restore OS biomarkers, serum antioxidants, and the glutathione redox balance (reduced glutathione/oxidized glutathione ratio) to levels comparable with that of the control group, particularly at dosage of 50 mg geraniin. Geraniin was not toxic to the HFD rats but exhibited protection against glucotoxicity and lipotoxicity particularly in the pancreas of the obese rodents. It is suggested that geraniin has the pharmaceutical potential to be developed as a supplement to primary drugs in the treatment of obesity and its pathophysiological sequels.

## Introduction

Excessive dietary intake of fat has long been linked to obesity (1) which has currently assumed alarming proportions as a global public health concern due to its attendant risks for human health and wellbeing. Chronic obesity and the accompanying deposition of fat in the tissues lead to a range of metabolic disorders, in particular, development of insulin resistance (IR) and type-2 diabetes mellitus. While some role of genetic predisposition for both obesity and diabetes is recognized, dietary composition has also been linked to the pathogenesis of IR, particularly a high intake of dietary fats (1, 2).

A link between high-fat diet (HFD) and oxidative stress (OS) has been recognized for long (3–6). It has been suggested that long-term feeding of a high-saturated fat diet acts as an inducer of OS, since it significantly attenuates the hepatic enzyme antioxidant system, and increases the levels of lipid peroxidation (LPO) products in the liver and plasma (5, 6). Other reports suggest a role for the OS in pathogenesis of metabolic derangements leading to IR and obesity (7) and diabetes mellitus (8).

It has been argued that an increase in OS precedes the development of obesity and metabolic derangements that are induced by an HFD (7) and as such it would be conceivable that amelioration of the elevated OS at this stage could possibly prevent or limit the extent of the subsequent metabolic perturbations and its sequelae. OS as well has been shown to play a major role in organ pathophysiology and is associated to organ dysfunctions (9, 10).

Plant-derived polyphenols and polyphenol metabolites have long been recognized for its prominent antioxidative benefits, with relatively minimal adverse actions. Antioxidant therapies with phytochemicals have been shown to be effective in suppressing multiple OS pathways, particularly in relation to obesity and its pathophysiological sequels (11).

**Figure 1:**
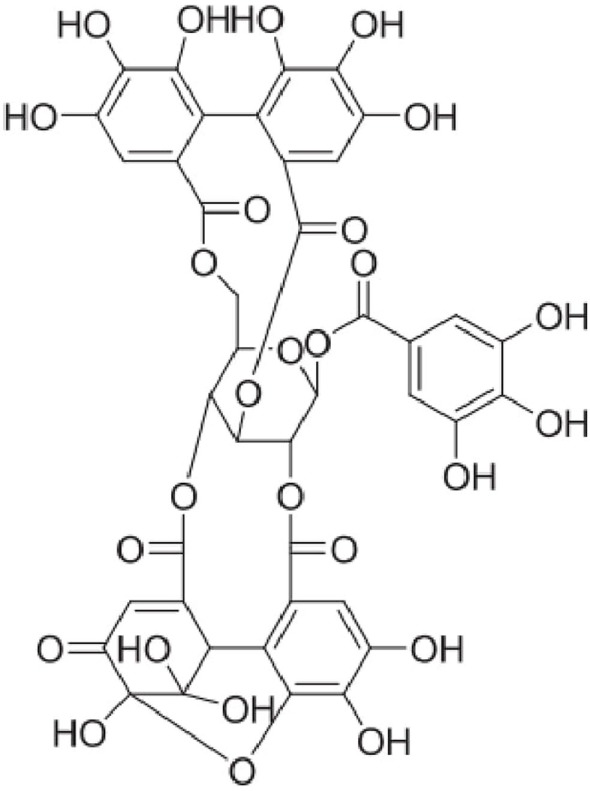
Chemical structure of geraniin.

Indigenous to Southeast Asia, *Nephelium lappaceum* L. (“rambutan” in Malay language), belongs to the family *Sapindaceae*. The fruit is taken fresh, canned, or processed. The dried fruit rind has been used as a natural health remedy for centuries (12). The major bioactive phenolic compounds from *N. lappaceum* L. rind have been identified as corilagin, elaeocarpusin, and geraniin (13). Palanisamy et al. (14) reported that among plants studied thus far, the highest yields of geraniin (up to 35%) can be extracted from the dried fruit rind of *N. lappaceum*. Geraniin (Figure 1) is also present abundantly in a wide variety of plant-based medicines and foods ranging from Chinese, Unani, and ayurvedic medicinal herbs such as *Phyllanthus niruri* (15), *Geranium thunbergii* (16), and *G. sibiricum* (17). Geraniin’s distribution, abundance, and bioactivity have been well documented in several reviews (18, 19).

*In vitro* studies have shown that geraniin exhibits high-antioxidant activity and exerts cytoprotective effect against cellular OS (13, 14, 17, 20). Recently, we showed that geraniin provided significant protection against metabolic dysfunctions induced by an HFD in rats and was not toxic at the dosages up to 50 mg/kg (21). It is possible that these protective effects of geraniin in HFD-fed rats could be related to its antioxidant effects. The present study seeks to evaluate the effect of geraniin administration on HFD-induced disturbance of redox homeostasis and organ toxicity in rats.

## Materials and Methods

### Materials, Reagents, and Instruments

Reverse-phase C18 silica (particle size 50 lm, pore size 60 Å) (Davisil; 633NC18E) (Grace, USA). Ethanol (absolute and denatured) (Scharlau Chemicals, Spain), Acetonitrile and dichloromethane (Mallinckrodt Chemicals, USA), Methanol and trifluoroacetic acid (Merck, Germany), and formic acid (Sigma Aldrich, USA). High-fat pellet (HFD, 60% fat by weight, AIN93G specifications purified diet) was purchased from Specialty Feeds Inc. (Glen Forrest, Western Australia), normal rat chow [normal diet control (ND), 5% minimum crude fat content, contained 25 mg/kg diet vitamin E of which 16.5 mg/kg diet was tocopherols, and 8.6 mg/kg diet was tocotrienols] was purchased from Gold Coin (Kuala Lumpur, Peninsular Malaysia), rat ELISA kits of advanced glycation end-product (AGE), malondialdehyde (MDA), protein carbonyl content (PCC), total antioxidant capacity (TAC), catalase (CAT), superoxide dismutase (SOD), total glutathione (TGSH), reduced glutathione (GSH), oxidized glutathione (GSSG), glutathione reductase (GR), and glutathione peroxidase (GPx) were purchased from Cayman (MI, USA). Accumet^®^basic AB15 pH meter (MA, USA), BDRAA220S belly dancer shaker (NC, USA), Beckman Coulter Microfuge 22R Centrifuge (CA, USA), BIO-RAD benchmark plus microplate reader with microplate manager 5.2.1 software (CA, USA), Branson ultrasonic cleaner 3510E-MTH (CT, USA), Eppendorf bench top refrigerated centrifuge 5702R (Hamburg, Germany), Eppendorf multi-channel pipettes and tips (Hamburg, Germany), GMC-260 microcentrifuge II (Kyonggi, South Korea), Mettler Toledo AB135-S/FACT electronic balance (Delaware, Switzerland), Milli-Q advantage A10 ultrapure water purification system with Q-POD Milipak (MA, USA), SEAT airflow monitor EN14-175 fume hood (Verniolle, France), and VTX-3000L electric mixer (Tokyo, Japan). All other chemicals, reagents, and consumables were purchased from Becton, Dickinson, and Company (NJ, USA), Merck (NJ, USA), Millipore (MA, USA), Sigma-Aldrich (MO, USA), Terumo (Tokyo, Japan), and Vétoquinol UK Limited (Buckingham, UK) unless stated otherwise. Glass column (250 mm × 50 mm i.d.) with fritted glass filter (porosity 2 µm) and vacuum inlet (Favorit, PLT Scientific, Selangor, Malaysia). HPLC (Shimadzu, Kyoto, Japan), Chromolith Performance RP-18 (100 × 4.6 mm) column (Merck, Darmstadt, Germany).

### Preparation of Geraniin From *N. lappaceum* L. Rind

*Nephelium lappaceum* L. was obtained from Kuala Lumpur, Malaysia which was later authenticated by the Herbarium of the Forest Research Institute of Malaysia. Crude ethanolic extract of *N. lappaceum* L. rind was prepared as described previously by Palanisamy and co-workers. While geraniin was purified from the crude extract by means of reverse-phase chromatography. Briefly, crude extract of 20 g was dissolved 40 mL of distilled water and loaded onto glass column containing C18 silica. Elution was carried out first with water (300 mL), followed by step-wise gradient of water and acetonitrile. Purity of geraniin (>95%) obtained was confirmed using HPLC (22).

### Animals and Housing

Thirty-two male, post-weaning (3-week-old) outbred Sprague Dawley (SD) rats (*Rattus norvegicus*) were obtained from the Animal House of Monash University (Monash University, Peninsular Malaysia). Males were used to eliminate variations in metabolic outcomes induced by sex in addition to their vulnerability to the impacts of diet-induced obesity (DIO) (23). They were randomized into four groups of eight rats each and accorded a 7-day acclimatization period to diets and experimental conditions prior to this *in vivo* study. A minimum of eight rats for each treatment group was required to give a significant finding at *p* < 0.05 levels (24). Normal rat chow and distilled water (dH_2_O) were fed *ad libitum*. Body weights were recorded at the end of the acclimatization period, which are presented as initial weights in this study. Throughout the experiment, all SD rats were housed individually in cages with stainless steel lids and with *ad libitum* access to food and water. They were maintained on a 12 h light–dark cycle (lights on at 0800 h; lights off at 2,000 h) with controlled temperature (22 ± 1°C) and humidity (55 ± 10%) in the animal housing facility. The use and handling procedure of animals was approved by the Monash University Animal Ethics Committee according to the ethics approval (Approval Code: AEC: MARP/2011/021).

### Dietary Treatments

Following acclimatization, one group of rats was maintained on normal rat chow (ND) while three groups were given high-fat pellet diet (HFD) for 10 weeks (25). Water was allowed *ad libitum* to all groups. Food and water consumptions were monitored daily. Body weight and blood glucose were measured weekly. The two treatment groups, were fed geraniin 10 and 50 mg/kg body weight, respectively, in addition to HFD during weeks 7–10 (4 weeks).

### Experimental Design and Sampling

At the end of week 10, rats in all groups were fasted for 16 h prior to being sacrificed. The rats were anesthetized by intraperitoneal injection of ketamine (90 mg/kg body weight) and xylazine (10 mg/kg body weight) warmed at 37°C. Blood samples were collected through cardiac puncture, into sterile Red Top vacutainers containing clot activator gel and centrifuged at 15,000 × *g* for 15 min at 4°C. Serum samples obtained were aliquoted into sterile cryovials and stored at −80°C for measurements of fasting serum MDA, PCC, AGE, and TAC, enzymatic antioxidants (SOD, CAT, and GPx), non-enzymatic antioxidants (GSH and GR), TGSH and GSSG. The organs pancreas, liver, heart and aorta, kidney, and brain were promptly harvested after sacrifice and dissection completed within 30 min. All organs were washed in ice-cold phosphate-buffered saline (1×) to remove excessive blood clot and dried on absorbent paper to remove all remaining fluids before weighing on an electronic balance to obtain a constant weight. The absolute weights were recorded. All the organs were then examined macroscopically. The ratio of each organ to final body weight (relative organ weight expressed as % of body weight) was also determined (21).

### Histopathological Study

Each organ was placed in a sterile universal bottle with lid and fixed in 10% neutral buffered formalin (NBF). At the end of immersion period, the NBF-preserved organs were cut to approximately 2 mm in thickness and placed into sterile, labeled tissue cassettes before being processed in an automated tissue processor through timed intervals of ascending grades of ethanol, xylene, and finally paraffin. A suitable sized mold was selected to best fit each organ section. The tissue sample was placed in the mold and orientated until desired position was achieved. After that, the mold was left to cool before the firmly solidified wax block was unmoulded. Excess wax was trimmed off the wax block with a scalpel leaving at least 2 mm all around the tissue sample. The paraffin tissue blocks were kept on ice (0–4°C) for approximately 5–10 min after which the blocks were sectioned to 4 µm thick. Ribbons of each tissue section were fished onto sterile, labeled ground edge microscopic slides flooded with 30% alcohol in a cooling plate at optimal temperature −7.2°C. They were allowed to air-dry overnight at room temperature. These sections were placed on their edges and baked for 15 min at 64– - 65°C in the oven. They were then processed in the automated tissue stainer through timed intervals (45 min) of xylene, descending grades of ethanol, immersed in tap water, dehydrated in 95% ethanol, and absolute ethanol followed by de-waxing in xylene. The hematoxyline and eosin (H&E) slides were then mounted with Distrene-Plasticiser-Xylene (DPX), covered with glass cover-slips and subjected to photomicroscopic observation with a bright field microscope by the pathologist in a blinded way Histological grading of inflammation and congestion was scored as: “‘−” = no morphological changes in histology; “+” = mild; “++” = moderate; ‘+++” = severe morphological changes in histology.

### Quantification of Oxidative Damage Biomarkers

#### Measurement of Fasting Serum LPO

Lipid peroxidation in the SD rats was determined as the serum concentrations of rat MDA based on the conjugation of avidin-horseradish peroxidase (HRP) bound MDA to a monoclonal antibody specific for rat MDA. The amount of bound avidin-HRP conjugate produced was reversely proportional to the concentration of MDA and correspondingly the endogenous LPO activity which was quantified spectrophotometrically at 450 nm.

#### Measurement of Fasting Serum Protein Oxidation (PCO)

Protein oxidation in the SD rats was determined as the serum concentrations of rat protein carbonyl (PCC). Protein carbonyls react with 2, 4-dinitrophenylhydrazine to form a Schiff base and correspondingly the production of hydrazone. The amount of hydrazone produced was proportional to the PCC and correspondingly the endogenous PCO activity which was quantified spectrophotometrically at 360–385 nm.

### Quantification of Antioxidant Defense System

#### Superoxide Dismutase

Superoxide dismutase activity in the serum of SD rats was determined based on the reduction of tetrazolium salt to formazan in the presence of superoxide anions (O_2_^•−^) generated by xanthine oxidase, and hypoxanthine. The amount of O_2_^•−^ produced was measured colorimetrically with formazan as the dye, which upon reduction changes from colorless to a yellow color. Oxidation rate of tetrazolium salt to formazan by O_2_^•−^ was inversely proportional to the endogenous activity of SOD. One unit of SOD is defined as the amount of enzyme needed to exhibit 50% dismutation of superoxide anions at 25°C which was quantified spectrophotometrically at 440–460 nm.

#### Catalase

Catalase activity in the serum of SD rats was determined based on the peroxidatic reaction of CAT with methanol to produce formaldehyde in the presence of an optimal concentration of hydrogen peroxide (H_2_O_2_). Formaldehyde specifically forms a bicyclic heterocycle with 4-amino-3-hydrazino-5-mercapto-1,2,4-triazole (Purpald). The amount of formaldehyde produced was measured colorimetrically with purpald as the chromogen, which upon oxidation changes from colorless to a purple color. One unit of CAT is defined as the amount of enzyme that causes the formation of 1.0 nmol of formaldehyde per minute per milliliter at 25°C which was quantified spectrophotometrically at 540 nm.

#### Glutathione Peroxidase

Glutathione peroxidase activity in the serum of SD rats was determined indirectly by a coupled reaction with GR. GSSG produced upon reduction of H_2_O_2_ by GPx is recycled to its reduced state (GSH) by GR and NADPH. The oxidation of NADPH to NADP^+^ is accompanied by a decrease in absorbance at 340 nm. Under conditions in which the GPx activity is rate limiting, the rate of decrease in A_340_ was directly proportional to the GPx activity. One unit of GPx is defined as the amount of enzyme that catalyzes the oxidation of 1 nmol of NADPH per minute per milliliter at 25°C.

#### Glutathione Reductase

Glutathione reductase activity in the serum of SD rats was determined based on the NADPH-dependent reduction of GSSG to GSH. The oxidation of NADPH to NADP^+^ is accompanied by a decrease in absorbance at 340 nm. The rate of NADPH oxidation was directly proportional to the GR activity. One unit of GR is defined as the amount of enzyme that catalyzes the oxidation of 1 nmol of NADPH per minute per milliliter at 25°C.

#### GSH Concentrations

Reduced glutathione concentrations, exclusive of GSSG, in the supernatants of SD rats were calculated by subtraction of GSSG concentrations from TGSH concentrations (Eq. 1).

(1) [GSH, μM]=Total glutathione (μM)−oxidised glutathione (μM)

#### GSSG Concentrations

Oxidized glutathione concentrations, exclusive of GSH, in the supernatants of SD rats were achieved by derivatizing GSH with 2-vinylpyridine. The rate of colorimetric change of 5′5-dithio-*bis*-2-(nitrobenzoic acid) (DTNB) to 5-thio-2nitrobenzoic acid (TNB) at 405–414 nm in the presence of GR and GSH was directly proportional to the concentration of GSSG.

#### TGSH Concentrations

Total glutathione concentrations in the supernatants of SD rats were quantified as the combined glutathione activities of both GSH and GSSG. The sulfhydryl group of GSH reacts with DTNB to produce a yellow colored TNB. The mixed disulfide, GSTNB that is concomitantly produced, is reduced by GR to recycle the GSH and further produce more TNB. The rate of TNB production was directly proportional to this recycling reaction which was in turn directly proportional to the concentration of GSH. Absorbance of TNB was monitored spectrophotometrically at 405–414 nm at 25°C.

#### Estimation of GSH/GSSG

Reduced glutathione to GSSG ratio in the serum of SD rats was calculated (Eq. 2).

(2) GSH/GSSG Ration=Reduced glutathione (μM)/Oxidised glutathione (μM)

#### Total Antioxidant Capacity

Total antioxidant capacity of the serum of SD rats was assessed by evaluating the combined antioxidant activities of all its constituents in both aqueous- and lipid-soluble forms. Antioxidants inhibit the oxidation of 2,2′-Azino-di-[3-ethylbenzthiazoline sulfonate] (ABTS) to ABTS^⋅+^ by metmyoglobin. The amount of ABTS^•+^ produced was proportional to the antioxidant concentration which was monitored spectrophotometrically at 750 nm for less interference.

### Statistical Analysis

Data obtained were analyzed using the GraphPad Prism statistical software, version 5.0 (CA, USA). One-way ANOVA with Tukey’s *post hoc* test was used for comparisons between variables and *t*-test for pair wise comparison between the different groups. All data are presented as mean ± SEM unless otherwise indicated. Significance was assigned at *p*-value <0.05 (confidence interval 95%). The experimental groups, ND, HFD, HFD + 10 mg G and HFD + 50 mg G, are represented by superscripts a, b, c, and d, respectively. Means with different superscripts are significantly different at *p*-value <0.05 between groups. For histopathological grading, a minimum of eight rats for each treatment group was sufficient to produce a significant finding at *p* < 0.05 levels.

## Results

### Effect of High-Fat Feeding and Geraniin Treatment on Oxidative Damage Biomarkers

At the end of week 10, the effects of high-fat feeding and geraniin supplementation on oxidative damage biomarkers in SD rats were investigated. The concentrations of MDA, a natural product of LPO, in the fasting serum of SD rats were measured to investigate the *in vivo* effects of feeding with geraniin (Table 1). High-fat feeding resulted in a statistically insignificant (*p* > 0.05) decrease in MDA in the HFD group compared with the ND-fed rats. However, a significant decrease in fasting serum MDA concentration was consistently observed in the geraniin-treated groups compared with both the HFD and ND groups. Conversely, the magnitude of effect at the dosages tested, 10 and 50 mg/kg did not differ significantly.

**Table 1:** Effect of a 10 week high-fat feeding and 4-week-geraniin treatment on oxidative damage biomarkers; malondialdehyde and protein carbonyl content, of Sprague Dawley rats.

OS biomarkers	Groups			
	ND	HFD	HFD + 10 mg G	HFD + 50 mg G
MDA (nmol/mL)	2.142 ± 0.123^c,d^	1.984 ± 0.039^c,d^	1.408 ± 0.058^a,b^	1.378 ± 0.089^a,b^
PCC (nmol/mL)	5.909 ± 0.001^b,c,d^	6.988 ± 0.004^a,c,d^	5.227 ± 0.001^a,b^	4.773 ± 0.001^a,b^

*Means with different superscripts are significantly different at p-value <0.05 between groups. The experimental groups, ND, HFD, HFD + 10 mg G and HFD + 50 mg G, are represented by superscripts a, b, c, and d, respectively*.

*ND, normal diet control; HFD, high-fat diet control; G, geraniin treated*.

The fasting serum PCCs in SD rats were also measured (Table 1). HFD-fed rats demonstrated a significant increase in PCC compared with ND-fed rats, while geraniin treatment showed significant decrease in PCC. Inclusions of geraniin at 10 and 50 mg significantly lowered PCC by 23 and 30%, respectively, compared with the HFD-fed rats.

### Effect of High-Fat Feeding and Geraniin Treatment on Serum Antioxidants

The effect of HFD and geraniin supplementation on enzymatic antioxidants (SOD, CAT, and GPx) in SD rats were investigated (Table 2). Mean SOD activity in the HFD-fed group was significantly lower compared with the ND-fed. Four-week treatment with geraniin caused a significant increase in fasting serum SOD activity, compared with both HFD and ND-fed groups. The 50 mg/kg dose, in particular, exhibited fasting serum SOD activities approximately twofold higher than that observed in the ND-fed rats (Table 2). A statistically insignificant increase in CAT activity was observed in the HFD group, compared with the ND group. Geraniin treatments as well-increased CAT activities in both treatment groups, but none of these were at statistically significant levels.

**Table 2:** Effect of 10-week high-fat feeding and 4-week-geraniin treatment on serum antioxidants of the Sprague Dawley rats.

Serum antioxidants	Groups			
	ND	HFD	HFD + 10 mg G	HFD + 50 mg G
SOD (U/mL)	4.534 ± 1.036^b,c,d^	1.073 ± 0.893^a,d^	1.961 ± 0.618d	9.332 ± 0.680^a,b,c^
CAT (nmol/min/mL)	8.355 ± 3.202	11.225 ± 3.735	12.905 ± 5.116	15.100 ± 5.350
GPx (nmol/min/mL)	18.750 ± 1.041^b^	15.280 ± 0.890^a,c,d^	20.340 ± 1.319	22.240 ± 0.907^a,b^
GR (nmol/min/mL)	137.534 ± 0.005^b,c,d^	91.689 ± 0.000^a,c,d^	188.472 ± 0.00^a,b^	208.847 ± 0.000^a,b^
GSH (μM)	0.091 ± 0.007^b,d^	0.004 ± 0.003^a,c,d^	0.120 ± 0.003^b,d^	0.210 ± 0.000^a,b,c^

*Means with different superscripts are significantly different at p-value <0.05 between groups. The experimental groups, ND, HFD, HFD + 10 mg G and HFD + 50 mg G, are represented by superscripts a, b, c, and d, respectively*.

*ND, normal diet control; HFD, high-fat diet control; G, geraniin treated; SOD, superoxide dismutase; CAT, catalase; GPx, glutathione peroxidase; GR, glutathione reductase; GSH, reduced glutathione*.

Pertaining to GPx activity, the HFD group demonstrated significant reduction in activity compared with the ND group (Table 2). Four-week-geraniin treatment at 10 and 50 mg/kg, caused a significant increase in fasting serum GPx activity compared with the HFD and ND controls, particularly at the 50 mg/kg dose (Table 2).

The effect of high-fat feeding and geraniin supplementation on non-enzymatic antioxidants (GR and GSH) in SD rats are shown in Table 2. A significant decrease in both GR activity and reduced GSH was observed in the HFD group compared with the ND group. Geraniin treatments, 10 and 50 mg, significantly raised the fasting serum GR activities and reduced GSH compared with both HFD and ND groups. Geraniin supplementation at 50 mg/kg was seen to significantly increase the non-enzymatic antioxidants when compared with both the ND and HFD-fed rats.

### Effect of High-Fat Feeding and Geraniin on GSSG, TGSH, and GSH:GSSG Ratio

At the end of week 10, the *in vivo* effects of high-fat feeding and geraniin supplementation on GSSG and TGSH was determined while GSH:GSSG ratio was calculated. A significant increase in GSSG concentrations in the HFD control was observed, compared with the ND control (Table 3). Four-week-geraniin treatment brought about a significant decrease in GSSG concentration, in a dose-dependent manner, compared with the HFD group. Geraniin treatment reduced the concentrations of GSSG, and the concentration after the higher dose of geraniin was observed to be lower than that in the ND group (Table 3; *p* < 0.05). The dosage at 50 mg/kg saw a reduction in GSSG far lower than that in the ND-fed group.

**Table 3:** Effect of 10-week high-fat feeding and 4-week-geraniin treatment on fasting serum total glutathione concentrations, oxidized glutathione concentrations, and reduced glutathione/oxidized glutathione ratio of the Sprague Dawley rats.

	Groups			
	ND	HFD	HFD + 10 mg G	HFD + 50 mg G
GSSG (μM)	2.234 ± 0.000^b,d^	2.276 ± 0.006^a,c,d^	2.219 ± 0.003^b,d^	2.167 ± 0.003^a,b,c^
TGSH (μM)	2.325 ± 0.007^b,d^	2.280 ± 0.003^a,c,d^	2.339 ± 0.006^b,d^	2.377 ± 0.003^a,b,c^
GSH:GSSG	0.041^b,d^	0.002^a,c,d^	0.054^b,d^	0.097^a,b,c^

*Means with different superscripts are significantly different at p-value <0.05 between groups. The experimental groups, ND, HFD, HFD + 10 mg G and HFD + 50 mg G, are represented by superscripts a, b, c, and d, respectively*.

*ND, normal diet control; HFD, high-fat diet control; G, geraniin treated; GSH, reduced glutathione; GSSG, oxidized glutathione*.

As for TGSH concentrations, a significant decrease was observed in the HFD control compared with the ND group (Table 3). Geraniin treatments, 10 and 50 mg, significantly raised the TGSH concentrations compared with the HFD group, in a dose-dependent manner. The ratio of GSH:GSSG, an indicator of OS, was calculated and expectedly a similar trend was observed with the 50 mg/kg dosage exhibiting a ratio significantly higher than even the ND group.

### Effect of High-Fat Feeding and Geraniin Treatment on TAC

**Figure 2:**
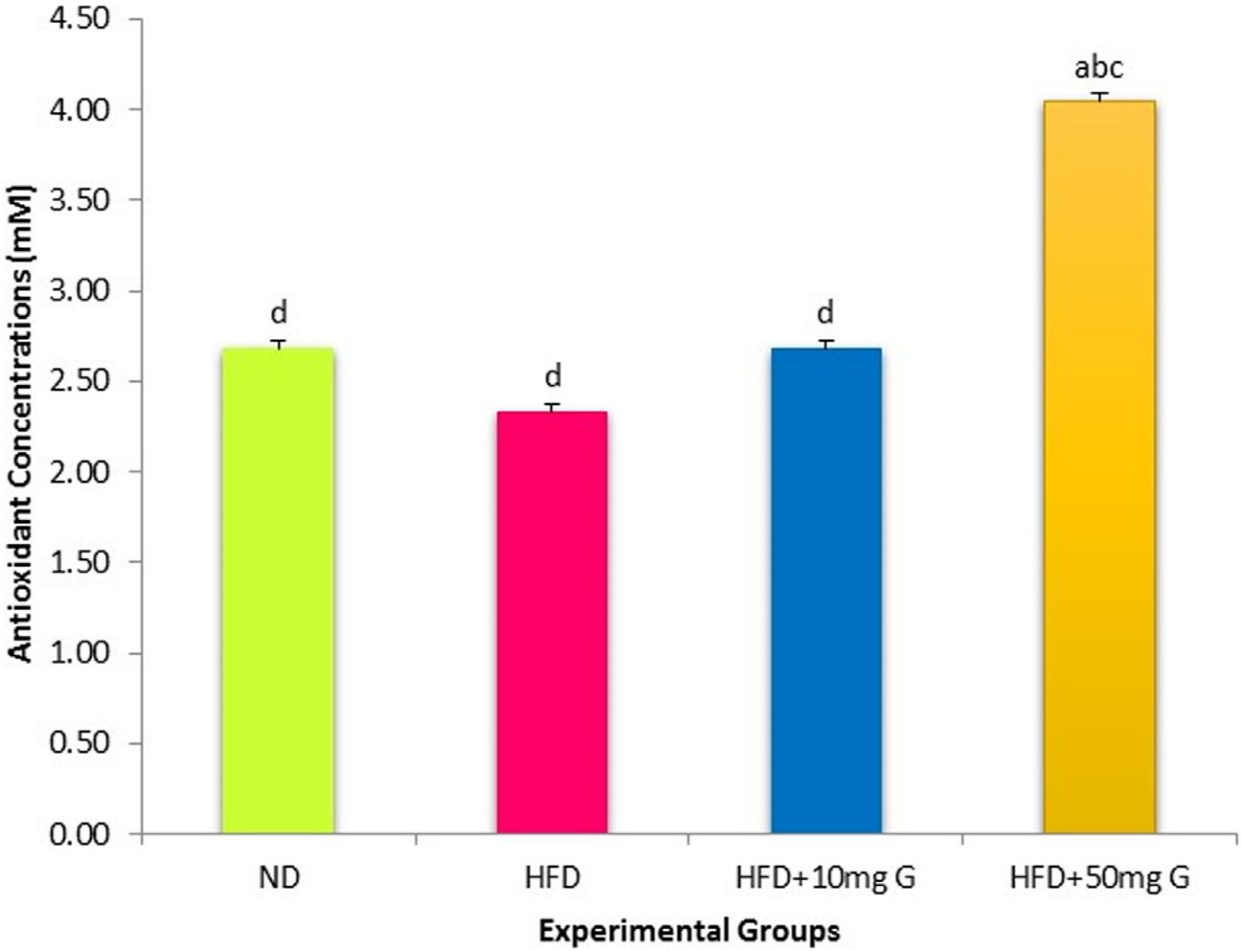
Effect of 10-week high-fat feeding and 4-week-geraniin treatment on fasting serum total antioxidant of the Sprague Dawley rats.

The TACs in SD rats were also estimated. The HFD-fed rats exhibited a small and statistically insignificant decrease in TAC compared with the ND-fed rats (Figure 2). However, 4-week-geraniin treatment, particularly at 50 mg inclusion brought about a significant increase in TAC compared with both HFD and ND controls.

### Histopathological Study

**Figure 3:**
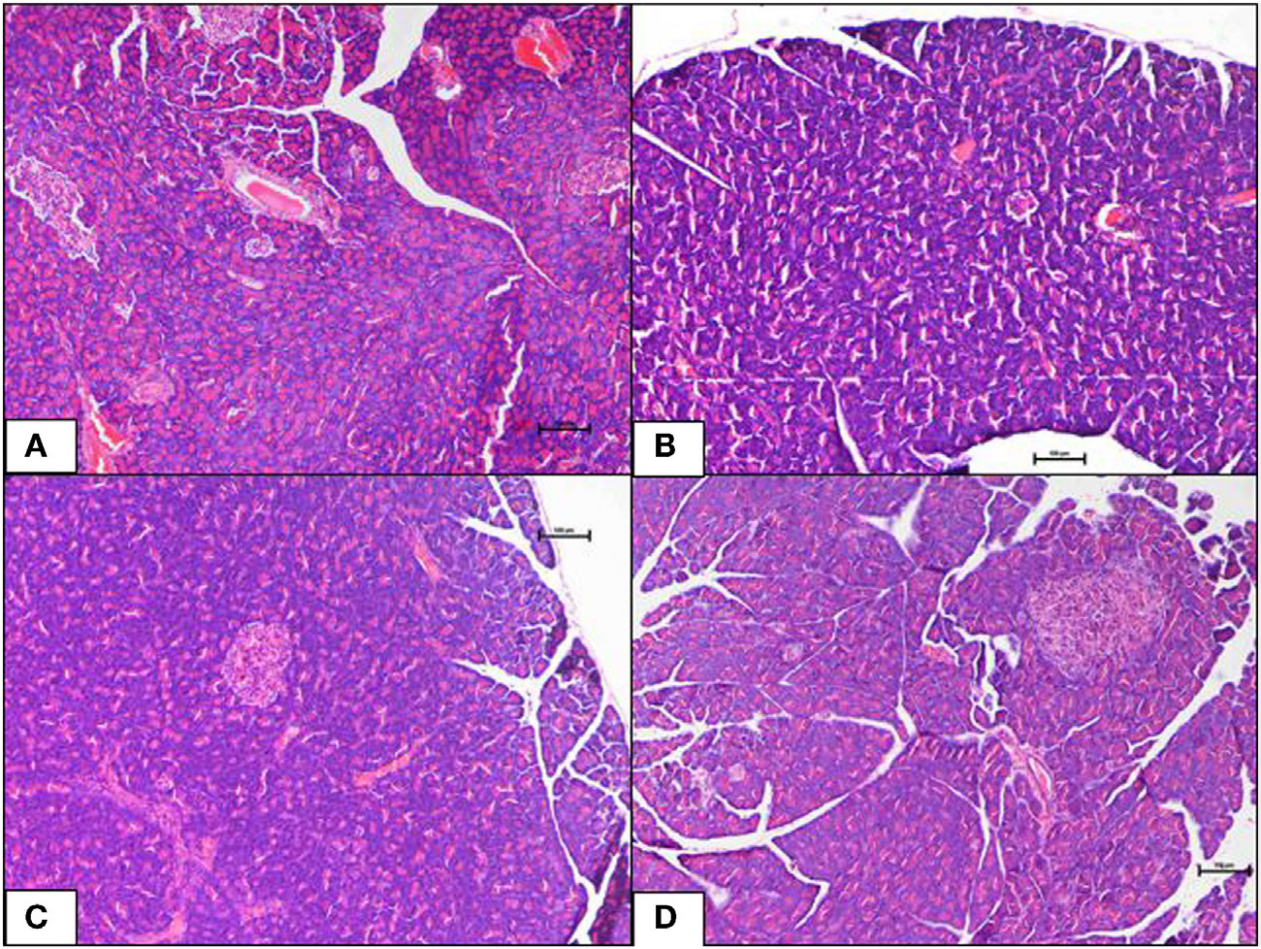
**(A–D)** A photomicrograph (hematoxyline and eosin, 100×) of the pancreas from the various experimental groups, **(A)** normal diet control: healthy pancreatic tissue, **(B)** high-fat diet: showing loss of architecture and focal necrosis, **(C)** 10 mg/kg geraniin: showing adequate healthy tissue and good regeneration, **(D)** 50 mg/kg geraniin: showing abundant regenerating acini and minimal evidence of damage. Scale bars represent 100 µm.

**Figure 4:**
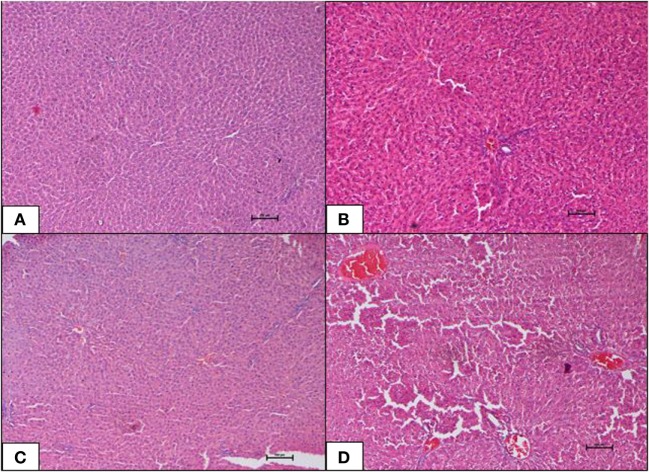
**(A–D)** Photomicrographs (hematoxyline and eosin, 100×) of the liver from the various experimental groups, **(A)** normal diet control, **(B)** high-fat diet, **(C)** 10 mg/kg geraniin, **(D)** 50 mg/kg geraniin, whereby **(A–C)** show normal hepatic architecture, and **(D)** shows focal central vein congestion and scattered periportal inflammation. Scale bars represent 100 µm.

**Figure 5:**
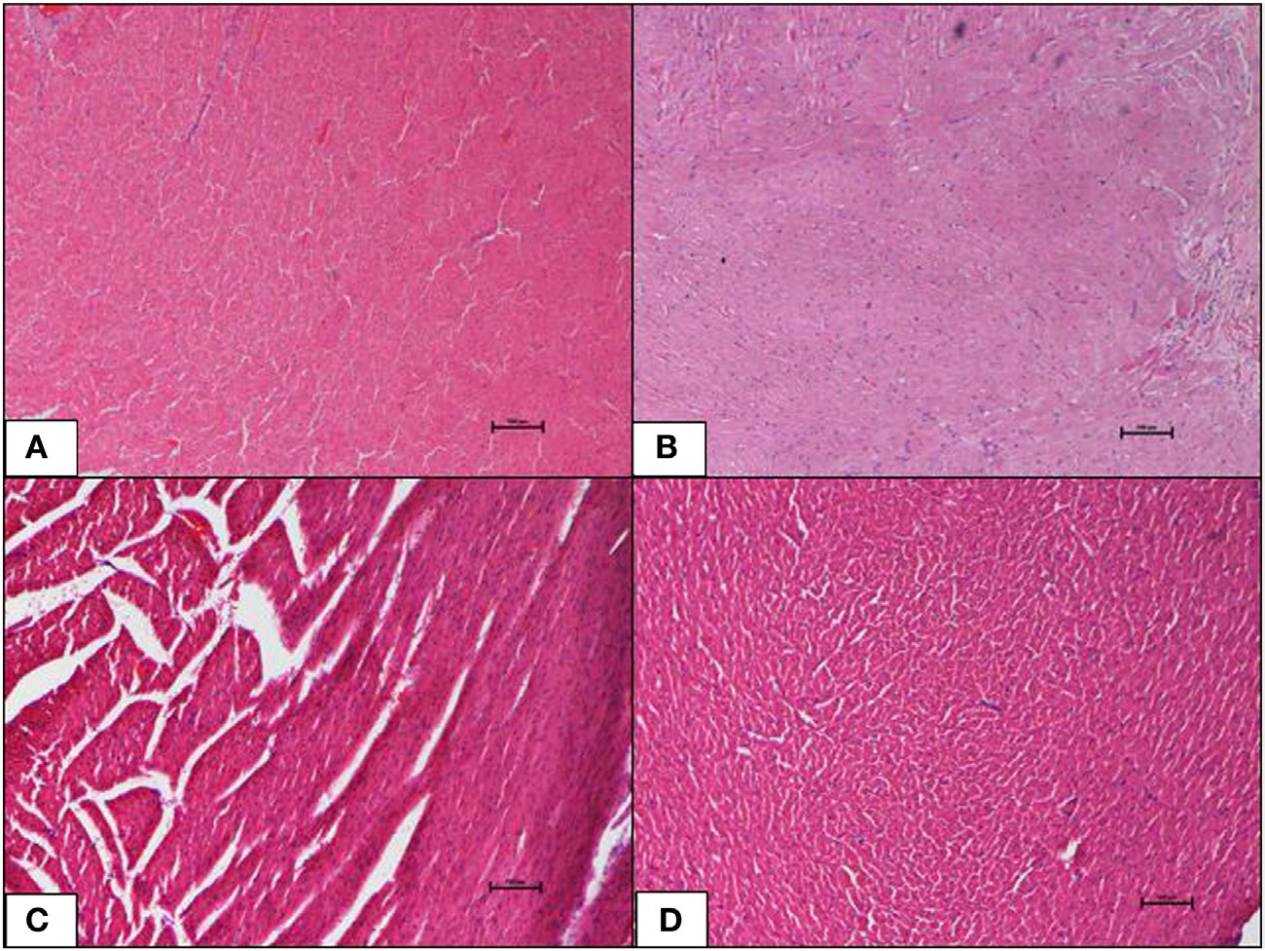
**(A–D)** Photomicrographs (hematoxyline and eosin, 100×) of the heart from the various experimental groups, **(A)** normal diet control, **(B)** high-fat diet, **(C)** 10 mg/kg geraniin, **(D)** 50 mg/kg geraniin, showing normal cardiac morphology in all groups, and no hypertrophy or necrosis observed. Scale bars represent 100 µm.

**Figure 6:**
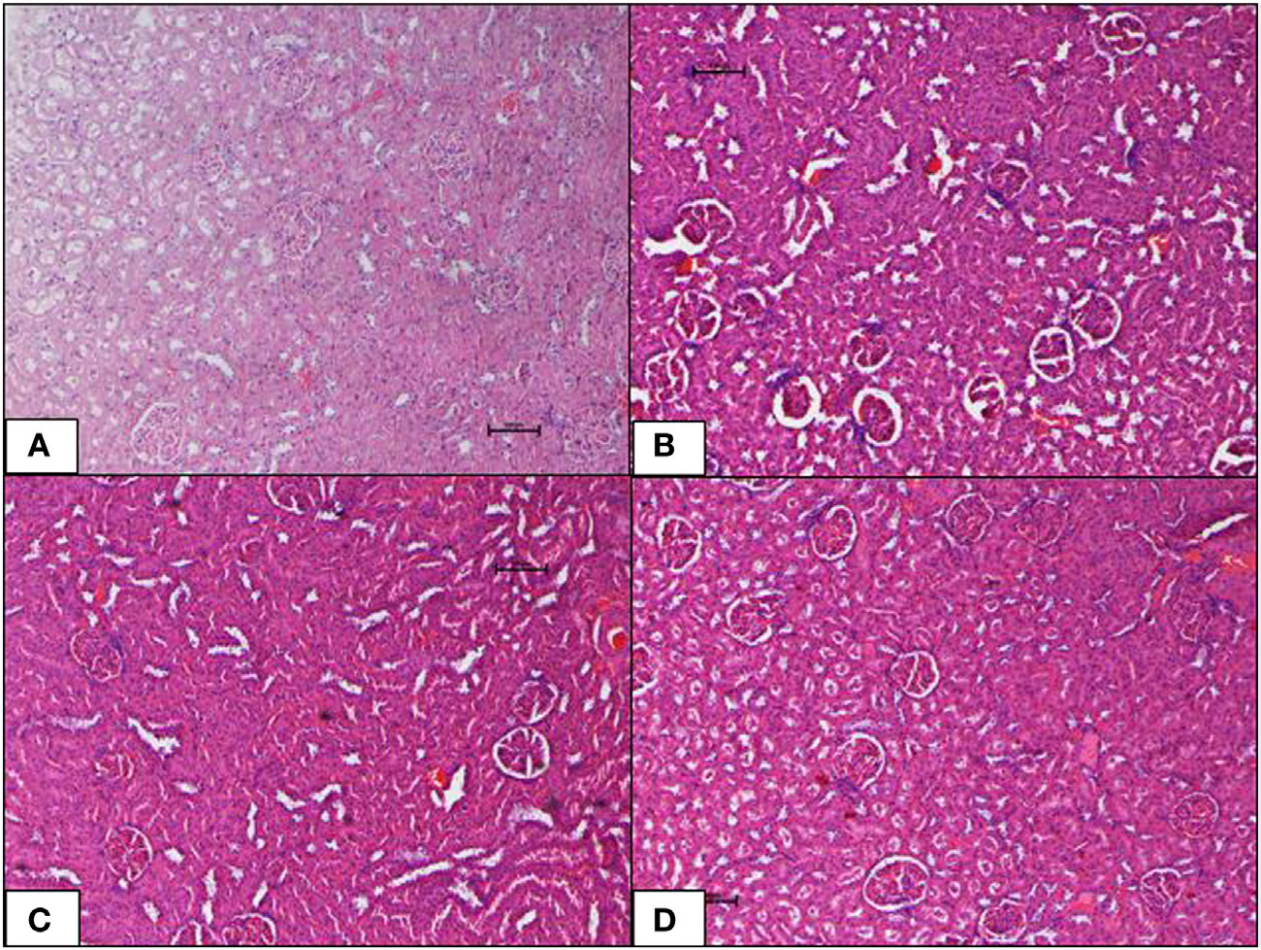
**(A–D)** Photomicrographs (hematoxyline and eosin, 100×) of renal tissue from the various experimental groups, **(A)** normal diet control, **(B)** high-fat diet, **(C)** 10 mg/kg geraniin, **(D)** 50 mg/kg geraniin, showing normal glomeruli and tubules and interstitium in all groups, with no evidence of inflammation or necrosis or toxic morphology. Scale bars represent 100 µm.

**Figure 7:**
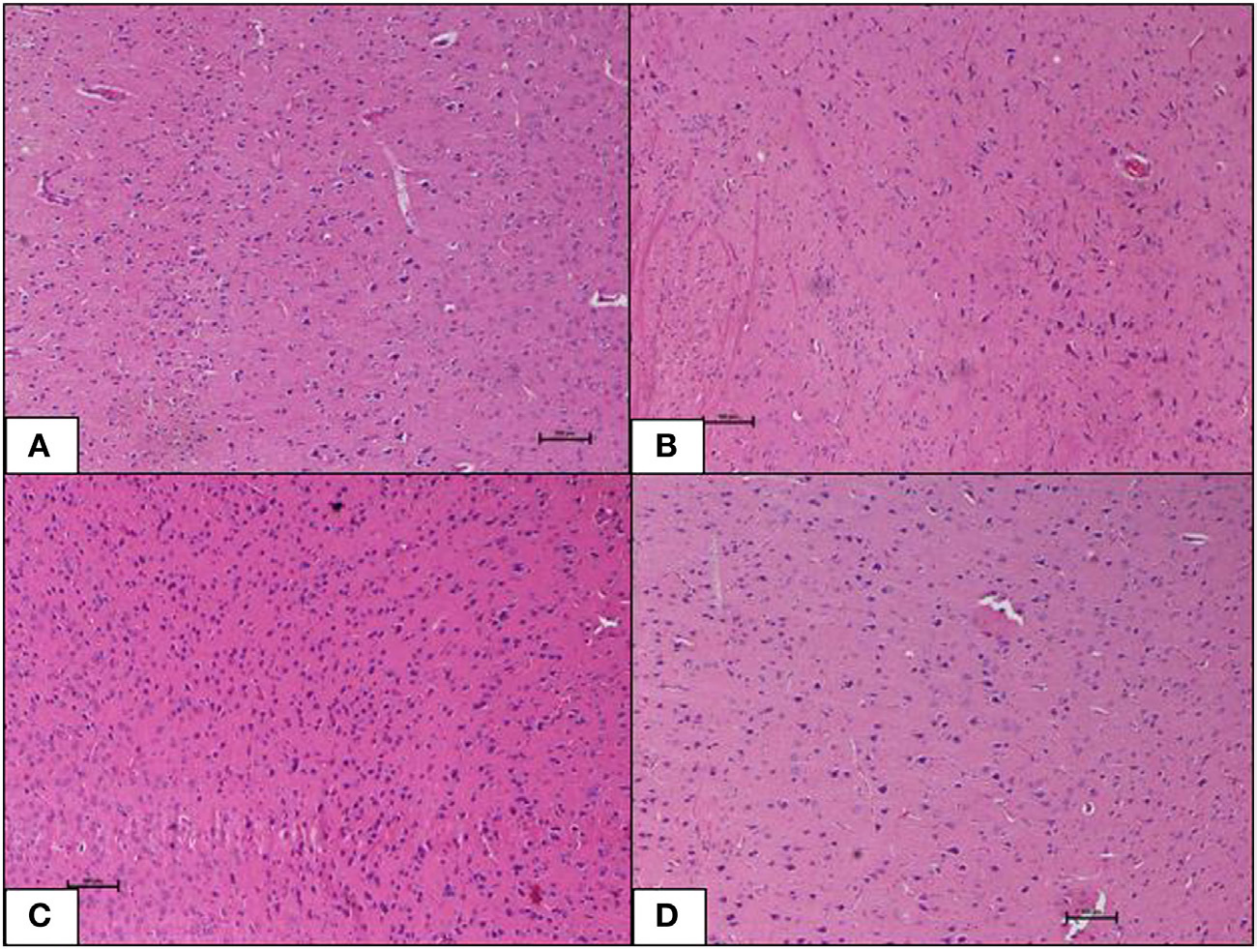
**(A–D)** Photomicrographs (hematoxyline and eosin, 100×) of the brain from the various experimental groups, **(A)** normal diet control, **(B)** high-fat diet, **(C)** 10 mg/kg geraniin, **(D)** 50 mg/kg geraniin, with all groups showing normal morphology and no inflammation, hemorrhage, or signs of toxicity. Scale bars represent 100 µm.

Histopathological changes in pancreas, liver, heart and aorta, kidney, and brain in all four groups were studied (Tables 1–5). The stained H&E slides were observed under the bright field microscope for expected morphological changes that included inflammation, congestion, and necrosis. Photomicrographs (Figures 3–7) were taken and grading for all the groups were recorded.

#### Pancreas

The ND control group showed healthy pancreas with normal exocrine acini and islets with adequate cellularity and distribution. There was no evidence of metabolic dysfunction-induced damage. On the contrary, the HFD control group showed morphological changes in pancreas suggestive of HFD-induced damage. Diffuse areas of damage to both the endocrine and exocrine parts were observed. There was loss of architecture and acini appeared necrotic in focal areas. The endocrine islets showed reduction in size, number, and distribution. Each islet itself had reduced number of cells. Some of the rats were also seen to have a total absence of islets in the pancreas (Figure 3B). Inclusions of geraniin at 10 and 50 mg brought about improvements in the pancreas compared with the HFD or ND controls (Figures 3C,D). Pancreas of the 10 mg geraniin-treated group showed areas of metabolic dysfunction-induced diffused damage, as found in the HFD-fed alone rats but focal areas of recovery was seen in the exocrine acini with normal acini seen in the periphery of the exocrine areas. However, there was no significant proliferation of the islets in terms of cellularity, number, and distribution. Pancreas of the 50 mg geraniin-treated rats showed only few residual areas of damage admixed with abundant areas of regenerating acini and good distribution of islets with healthy cellularity comparable with the ND-fed rats (Table 4; Figures 3A–D). Geraniin treatment, by histopathology, being moderate in 50 mg and mild in 10 mg, exhibits its novel antidiabetic potential on the pancreas.

**Table 4:** Effect of 10-week high-fat diet and 4-week-geraniin feeding on pancreas necrosis of the Sprague Dawley rats.

No	Groups			
	ND	HFD	HFD + 10 mg G	HFD + 50 mg G
1	−	+++	+	−
2	−	+++	++	+
3	−	+++	++	−
4	−	++	+++	−
5	−	+++	++	−
6	−	+++	+	+
7	−	++	++	−
8	−	+++	+	−

*ND, normal diet control; HFD, high-fat diet control; G, geraniin treated*.

*“−” = no morphological changes in histology; “+” = mild; “++” = moderate; “+++” = severe morphological changes in histology*.

#### Liver

Liver of the ND control group displayed normal architecture with normal central vein and well-spaced portal triads. Hepatocytes and sinusoids appeared normal. There was no evidence of congestion, inflammation, or necrosis. Interestingly, the HFD control group also showed normal liver histology as in the ND-fed rats with no indication of metabolic dysfunction-induced hepatic damage. The 10 mg geraniin-treated group also showed normal liver morphology with no congestion in the central vein or portal triads, as observed in the ND and HFD controls. There, however, was morphological change in the liver of 50 mg geraniin-treated group. This group displayed focal areas of congestion of central vein and periportal scattered inflammation (Table 5; Figures 4A–D).

**Table 5:** Effect of 10-week high-fat diet and 4-week-geraniin feeding on liver of the Sprague Dawley rats.

No	Groups											
	Liver congestion				Liver inflammation				Liver necrosis			
	ND	HFD	HFD + 10 mg G	HFD + 50 mg G	ND	HFD	HFD + 10 mg G	HFD + 50 mg G	ND	HFD	HFD + 10 mg G	HFD + 50 mg G
1	−	−	−	−	−	−	−	−	−	−	−	−
2	−	−	+	−	−	−	−	−	−	−	−	−
3	−	−	−	+	−	−	−	+	−	−	−	−
4	−	−	+	−	−	−	+	−	−	−	−	−
5	−	−	−	+	−	−	−	−	−	−	−	−
6	−	−	+	−	−	−	−	−	−	−	−	−
7	−	−	−	−	−	−	−	−	−	−	−	−
8	−	−	−	−	−	−	−	−	−	−	+	−

*ND, normal diet control; HFD, high-fat diet control; G, geraniin treated*.

*“−” = no morphological changes in histology; “+” = mild; “++” = moderate; “+++” = severe morphological changes in histology*.

#### Heart and Aorta

The ND control group showed normal cardiac morphology with myocytes well-arranged and aorta was normal. There was no evidence of hypertrophy or necrosis. The HFD control group also showed normal heart histology as in the ND-fed rats with no indication of metabolic dysfunction-induced cardiac damage. Inclusions of geraniin at 10 and 50 mg, neither caused cardiac toxicity nor induced morphological changes and showed normal cardiac morphology with well-arranged cardiomyocytes and normal lumen of aorta in the absence of hypertrophy or necrosis, as observed in the ND and HFD controls (Figures 5A–D).

#### Kidney

The ND control group showed healthy kidneys with normal glomeruli and tubules. There was no evidence of atrophy of glomeruli or casts in the tubules. Interstitium was normal. The HFD control group, interestingly, also showed normal kidney histology as in the ND-fed rats with no indication of metabolic dysfunction-induced renal damage. Inclusions of geraniin at 10 and 50 mg, neither caused renal toxicity nor induced morphological changes in the kidneys and showed normocellular glomeruli, tubules, interstitium, and architecture comparable with the HFD or ND controls (Figures 6A–D).

#### Brain

Brain tissue of the ND control group showed normal morphology with no inflammation, hemorrhage, or signs of toxicity. The neurons were healthy and well distributed. The HFD control group also showed normal brain morphology as in the ND-fed rats. Inclusions of geraniin at 10 and 50 mg, neither caused neurotoxicity nor induced morphological changes and showed normal brain morphology with well-distributed neurons in the absence of inflammation, hemorrhage, or signs of toxicity, as observed in the ND and HFD controls (Figures 7A–D).

## Discussion

Metabolic disturbances and IR caused by obesity have been linked to OS. Both in obese human subjects and experimental animals, obesity has been shown to be accompanied by an increase in OS markers (26–28). An augmented OS if coupled with an attenuated antioxidant capacity tends to disrupt the normal redox homeostasis leading to irreversible damage to membranes and other macromolecules (29, 30). HFD administration is routinely employed in experimental models of obesity and metabolic syndrome wherein the animal subjects, mostly rats or mice, display an increase in weight, IR, and dyslipidemic changes. It has been argued that excessive accumulation of fat leads to enhanced production of ROS in adipocytes and systemic tissues (27). Obesity, IR and hyperglycemia, develop over a period of several weeks of HFD administration and it has been demonstrated that increased OS precedes these changes (7). Addressing these elevated levels of stress is therefore likely to afford protection against or amelioration of the metabolic consequences of an HFD.

In the present study, rats were fed an HFD for 10 weeks. Two treatment groups, geraniin either at 10 mg or 50 mg/kg body weight per day, was administered from weeks 7–10. At the end of 10 weeks, markers of OS, antioxidant defense, and the histopathological changes were investigated. A group of rats on standard diet (ND) for the same period served as the negative control while another group on HFD for 10 weeks (without geraniin) served as the positive control.

In the HFD group, among the markers of oxidative damage tested, there was a significant increase in PCC, indicative of oxidative damage to proteins, and a statistically non-significant decrease in MDA levels (LPO) compared with the ND. The effect of HFD on enzymatic antioxidant defenses; saw a statistically significant decrease in SOD and GPx, and a statistically non-significant increase in CAT compared with the ND. Similar observations of raised PCC with a corresponding decrease in enzymatic antioxidants (SOD and GPx) have been reported in number of studies on an HFD (6, 7, 31, 32). Our studies, however, did not show a significant rise in MDA or a reduction in CAT, which are also indicators of OS. A reason for this could be that our analyses were performed using plasma as opposed to that of tissue homogenates by others (33).

The antioxidant defenses of the glutathione pathway were also significantly altered in HFD as compared with ND group, indicating the presence of OS. The OS markers; reduced GSH, GR, and TSSG were significantly decreased, while oxidized GSH (GSSG) was significantly raised, with a consequent decrease in the GSH:GSSG ratio in the HFD group. In general, HFD was accompanied by increased OS, characterized by the reduction in antioxidant enzymes (SOD, GPx, and GR) and GSH levels that correlate with the increase in PCC and GSSG. However, the oxidative imbalance observed in HFD group, through the above markers, was not reflected in the measurement of its TAC. It can be assumed that isolated measurement of TAC does not give a clear picture OS (33) and that both the glutathione system as well as the enzymatic antioxidant will need to be profiled and studied together.

Geraniin was administered after a 6-week HFD feed for a period of 4 weeks, at the dosages of 10 or 50 mg/kg body weight. While HFD by itself did not induce significant changes in LPO products, geraniin treatment produced a significant, dose-dependent decrease in MDA in HFD-fed rats, to levels even below than those seen in the negative controls (ND group). Likewise, PCC, which was raised by HFD, was also significantly reduced in a dose-dependent manner by geraniin administration. Several studies have reported geraniin’s antioxidant potential through *in vitro* assays (13, 20, 34, 35). Our study, for the first time, demonstrates its antioxidant potential in an *in vivo* situation.

Geraniin’s effect on serum antioxidants (SOD, CAT, and GPx) was more pronounced at the higher dosage, particularly for SOD. The most marked effect of geraniin treatment was evident on the glutathione antioxidant pathway, indicating its protective role against excessive generation of oxidative molecules. The glutathione pathway represents the scavenging system of formed free radicals. The concentration of GPx, GR, and TGSH was significantly raised with geraniin treatment and at the higher dosage its levels had surpassed that of the ND group. There was also a statistically significant dose-dependent decrease in GSSG levels, which correlated with the increase in GSH:GSSG levels. The overall effect of geraniin treatment, seem to suggest that its actions against HFD-induced OS is largely due to its ability to scavenge reactive oxygen species. While there are a number of reports on geraniin’s *in vitro* free radical scavenging ability (13, 20, 34, 35). Aayadi et al. (36) recently suggested that geraniin’s free radical scavenging ability conferred mice protection against hepatotoxicity.

It was postulated that in an HFD there is an early upregulation of expression of genes involved in ROS production, in liver, and adipose tissues, while insufficient upregulation of GPx production in the liver (7, 27). This makes it susceptible to selective oxidative damage and subsequent development of IR and obesity. In our study, we found a suppression of the glutahione mediated scavenging action at 10 weeks of administering HFD, which was antagonized by concomitant administration of geraniin during the last 4 weeks of HFD administration. We therefore speculate that geraniin supplementation has a protective effect against HFD-induced IR and obesity by restoring the redox homeostasis. The duration of the present study was only for 10 weeks and this is a limitation and requires further, longer duration studies to test the above contention.

Geraniin was established to exert significant protective effects on metabolic derangements in a DIO rodent model (21). However, research into its toxicity or adverse reactions, particularly on vital organs which are recognized as the target sites of dietary phenolics is lacking, let alone in a DIO rodent model. In view of that, this study firstly established a DIO rodent model using male SD rats induced by prolonged high-fat feeding (HFD, 60% fat by weight; 10 weeks), followed by the evaluation of geraniin’s protective potential on organs at doses achievable in this DIO rodent model.

In our earlier study, we showed that prolonged high-fat feeding resulted in DIO, particularly visceral obesity in SD rats. The HFD-fed rats also had significantly higher weights of pancreas, liver, and heart and aorta when compared with the ND-fed rats. No significant differences were observed in the weights of kidneys and brains across all four groups. Four-week-geraniin treatment, particularly at 50 mg, significantly brought about a significant weight reduction in the pancreas, liver, and heart and aorta of the obese rats (21).

In this study, we present the histopathological observations of the organs examined; pancreas, liver, heart and aorta, kidney, and brain. HFD-fed group displayed severe necrosis in the pancreas (Table 4; Figure 3B). When a high-caloric, energy-dense diet is continually consumed, a positive energy balance is perpetuated. This results in excessive energy storage particularly in the form of triglyceride (TG) in the pancreas. TGs are ectopically deposited as microlipid droplets in the cytosol and near the cell membrane in the beta-cells in fatty pancreas (37). The obese rats hence developed lipotoxicity in the pancreas (Table 4; Figure 3B). Accretion of TG tends to impose a higher-pressure load on the pancreas and thus, compresses it causing intracellular architecture loss, which ultimately results in severe pancreatic necrosis in the HFD-fed group. A similar outcome was reported Catalano et al. (38), where a high-caloric diet-induced IR and type-2 diabetes mellitus (T2DM) through ectopic fat deposits in pancreas of rats.

Severe pancreatic necrosis in the HFD-fed rats may have impaired glucose metabolism in these rats. The ruptured or totally absent endocrine islets of Langerhans, decreased beta-cell population, and exocrine pancreatic cell damage (Figure 5B) observed will significantly challenge beta-cell survival. Consequentially, reduced beta-cell survival will disrupt normal beta-cell function and inhibit insulin biosynthesis and secretion in the HFD-fed rats. The above observation corroborate with findings reported by Lingohr et al. (39), Qiu et al. (40), and Ou et al. (41). Beta-cell survival is pivotal in determining the endocrine islet area and function because beta-cell is the only insulin-producing cell type in pancreas (serves as a glucostat) that strictly regulates glucose threshold. Furthermore, abnormalities in exocrine pancreas have been associated with various degrees of diabetogenesis (40). Findings related to pancreatic pathology obtained in the current investigation have provided strong evidence that obesity-induced T2DM was successfully established in this DIO rat model. Previous findings have reported similar observations (37, 42, 43).

In our earlier study, we reported that 4-week-geraniin treatment significantly mitigates anomalies in fasting plasma glucose, serum insulin, IR, and pancreas weight (21). Histopathological evaluation in the present study confirms geraniin’s potential, in a dose-dependent manner, to regenerate pancreatic cells and protect the pancreas against obesity-induced T2DM. Our results provide evidence that geraniin might be useful in the treatment of non-alcoholic fatty pancreas disease in obese subjects.

Liver of the HFD-fed group did not show obesity-induced hepatic damage. Geraniin supplementation at 10 mg/kg as well did not display any hepatic damage (Table 5; Figure 4C). However, mild hepatic morphological changes, appearing as scattered inflammation, were observed in the 50 mg geraniin-treated rats (Table 5; Figure 3D). Over-nutrition in rodents as a model of non-alcoholic fatty liver disease (NAFLD) induced by a high-caloric diet has been prevalently used owing to the strong link between fatty liver formation and obesity. However, in a number of studies (44, 45), rodent models failed to develop a full spectrum of histological evidence of NAFLD despite evolving many pathophysiological changes typical of obesity. A similar observation was obtained in this study. Liver injury, in fact, is a complex process involving both parenchymal and non-parenchymal cells resident in liver and also involves the recruitment of other cell types to liver in response to the degree of inflammation, fibrosis, steatosis, and necrosis (46–48).

Geraniin supplementation did not cause hepatic toxicity at the lower dose of 10 mg but a mild damaging effect on liver causing hepatic changes at the higher dose (50 mg) was observed (Table 5; Figure 4D). Interestingly, 4-week-geraniin treatment at this dose (50 mg) has been shown to significantly mitigate biomarkers of liver dysfunction (ALT and AST) and the liver weight (21), suggesting that geraniin although showing mild hepatic damage has therapeutic potential. Further to this, several studies have reported geraniin’s hepatoprotective ability against carbon tetrachloride (36), ethanol (49), and thioacetamide (50).

However, it may not yet be a suitable mode of therapy until further preclinical trials are conducted. It is potentially useful as a supplement to primary drugs to lower NAFLD risk in the treatment of obesity. The mild condition obtained in liver histopathology further raises the prospect that the development of NAFLD may require a longer induction time in addition to genetic modifiers present in the outbred SD rats that resist the ectopic progression of NAFLD.

In this study, H&E stained sections of heart, kidney, and brain did not show morphological changes or diet-induced damage in the HFD-fed rats. Previous studies have highlighted the possibility that obesity-induced deformities in cardiovascular system of DIO rodent models varied over diet induction lengths (51, 52). These studies thus collectively raised the prospect that fat feeding (8–16 weeks) in rodents did not result in the full spectrum of cardiovascular dysfunctions despite evolving metabolic derangements associated with macrovascular complications in the face of obesity. The development and progression of macrovascular diseases might thus require a prolonged diabetic duration (i.e., many years and often decades). Outcomes as such are in line with observations in this study.

Previous studies also showed that DIO rodent models that depict the metabolic contexts of diabetic renal complications in humans possibly produce variable outcomes with regards to the degree of congestion, inflammation, atrophy, and necrosis (53). In addition, a few studies have suggested that diabetic renal complications induced by an HFD are a cumulative derangement over a longer time course (54, 55).

Obesity and its clinical manifestations have been recognized as the key players of neuropathophysiology (i.e., vascular dementia; Alzheimer’s disease) and neurological co-morbidity including psychiatric disorders such as depression (56, 57). In this study, brain histopathology did not reveal any morphological changes or diet-induced damage in the HFD-fed rats.

Geraniin supplementation, at the doses used, did not cause toxicity to the heart, kidney, or brain of the rodents (Figures 5 and 7A–D). In fact, geraniin, at a dose of 20 mg/kg injected intraperitoneally, was recently shown to ameliorate cisplatin-induced nephrotoxicity in mice by inhibiting OS and inflammatory response (58).

Geraniin has also been reported to exhibit neuroprotection in an *in vitro* study. In this study, hindered by the inability to induce neurodysfunction in the DIO rodent model, it was not possible to show any protective potential of geraniin on brain. A more detailed study would be required to further investigate this. The present study, however, provides pioneering *in vivo* evidence for protective and regenerative effects of geraniin on the pancreas in DIO rodent models. Further, geraniin treatment by itself does not appear to produce any major toxicity in vital organs, in the doses employed in this study (59).

## Conclusion

Geraniin, a polyphenol compound derived from *N. lappaceum L*. was observed to attenuate OS induced by an HFD, by restoring the redox homeostasis. This study is the first to the best of our knowledge, to show that geraniin was able to restore OS biomarkers, serum antioxidants, and the glutathione redox balance to levels comparable with that of the control group. In doing so, geraniin can be of value in preventing HFD-induced development of IR, obesity, and metabolic derangements including diabetes mellitus. Longer term studies are strongly desirable to explore the potential value of this plant compound as a dietary oral supplement for the prevention of metabolic derangements such as IR, obesity, diabetes mellitus, and its complications, that may be linked to the OS produced by a faulty diet.

Protective assessment of geraniin against HFD-induced changes has never been investigated in rodents or any other *in vivo* model, particularly on recognized target sites of dietary phenolics and their metabolite derivatives. This study, to the best of our knowledge, is the first to provide pioneering *in vivo* evidence of DIO in a rodent model using male SD rats. In this study, 10-week of high-fat feeding was shown to sufficiently induce obesity in the SD rats having metabolic anomalies typical of DIO, particularly evident in the pancreas. In a previous study, the obese rats demonstrated both IR and beta-cell dysfunction (21). Together, these results collectively proposed the development of T2DM in the obese rats. Oral geraniin treatment at doses achievable *in vivo*, being moderate in 50 mg and mild in 10 mg, exhibited its novel therapeutic potential by protecting the pancreas against glucotoxicity and lipotoxicity induced by an HFD. Geraniin treatment by itself, as evident from histopathology, did not cause toxicity in heart and aorta, kidney and brain of the DIO rodent model, although mild changes in the liver were observed at the higher dose. Geraniin therefore has the pharmaceutical potential to be developed as a supplement to primary drugs in the treatment of obesity and its pathophysiological sequels. However, the compound may not yet be a suitable principal mode of therapy until further preclinical trials are conducted. Furthermore, thorough investigation of its acute and chronic toxicity in normal animals, studies on its mode of action, bioavailability, and pharmacokinetics are recommended.

## Ethics Statement

The use and handling procedure of animals was approved by the Monash University Animal Ethics Committee according to the ethics approval (Approval Code: AEC: MARP/2011/021).

## Author Contributions

The authors’ responsibilities were as follows—UP designed the study, supervised the research, and prepared the final version of the paper, AC carried out the research and wrote the draft manuscript, SG aided in the design and edited the manuscript, SC carried out the histopathological interpretation while MM contributed to the design of manuscript and helped edit it. All authors: read and approved the final manuscript.

## Conflict of Interest Statement

The authors declare that the research was conducted in the absence of any commercial or financial relationships that could be construed as a potential conflict of interest.
